# Oral lichen planus: comparative efficacy and treatment costs—a systematic review

**DOI:** 10.1186/s12903-022-02168-4

**Published:** 2022-05-06

**Authors:** Shaiba Sandhu, Brittany A. Klein, Malak Al-Hadlaq, Prazwala Chirravur, Amal Bajonaid, Yuanming Xu, Rossella Intini, Mai Hussein, Piamkamon Vacharotayangul, Herve Sroussi, Nathaniel Treister, Stephen Sonis

**Affiliations:** 1 Division of Oral Medicine and Dentistry, Brigham and Women’s Hospital/ Dana Farber Cancer Institute, Boston, USA; 2 Department of Oral Medicine, Infection and Immunity, Harvard School of Dental Medicine, Boston, USA; 3 Harvard Medical School, Boston, MA USA; 4 Ministry of Health and Population, Cairo, Egypt; 5 Present Address: Department of Oral and Maxillofacial Surgery, Massachusetts General Hospital, 55 Fruit Street, Boston, MA 02114 USA

**Keywords:** Oral lichen planus, Treatment, Cost, Efficacy, Critical review

## Abstract

**Objective:**

To compare the reported efficacy and costs of available interventions used for the management of oral lichen planus (OLP).

**Materials and methods:**

A systematic literature search was performed from database inception until March 2021 in MEDLINE via PubMed and the Cochrane library following PRISMA guidelines. Only randomized controlled trials (RCT) comparing an active intervention with placebo or different active interventions for OLP management were considered.

**Results:**

Seventy (70) RCTs were included. The majority of evidence suggested efficacy of topical steroids (dexamethasone, clobetasol, fluocinonide, triamcinolone), topical calcineurin inhibitors (tacrolimus, pimecrolimus, cyclosporine), topical retinoids, intra-lesional triamcinolone, aloe-vera gel, photodynamic therapy, and low-level laser therapies for OLP management. Based on the estimated cost per month and evidence for efficacy and side-effects, topical steroids (fluocinonide > dexamethasone > clobetasol > triamcinolone) appear to be more cost-effective than topical calcineurin inhibitors (tacrolimus > pimecrolimus > cyclosporine) followed by intra-lesional triamcinolone.

**Conclusion:**

Of common treatment regimens for OLP, topical steroids appear to be the most economical and efficacious option followed by topical calcineurin inhibitors. Large-scale multi-modality, prospective trials in which head-to-head comparisons interventions are compared are required to definitely assess the cost-effectiveness of OLP treatments.

**Supplementary Information:**

The online version contains supplementary material available at 10.1186/s12903-022-02168-4.

## Introduction

Oral lichen planus (OLP) is a chronic, T-cell-mediated inflammatory condition, with a global prevalence between 0.1 and 3.2% [1, 2]. It is most common in the fourth-fifth decade of life and has a female predilection [1]. Clinically, OLP is characterized by white reticulations (Wickham striae), erythema, and/or ulcerations. While there is no consensus on subtypes, OLP is often categorized as reticular/keratotic, erythematous/erosive, or ulcerative. OLP can be either asymptomatic or symptomatic, and when symptomatic, can range from mild sensitivity to significant pain that impacts quality of life. OLP is considered an oral potentially malignant disorder with a malignant transformation rate of 0.4–1.4% [3].

The exact etiology of OLP is unknown, and there is currently no known cure [2]. The primary therapeutic goal is symptom management and current treatment options include corticosteroids, calcineurin inhibitors, retinoids, photodynamic therapy, and natural alternatives, although with varying degrees of efficacy [4, 5]. A recent meta-analysis of 55 RCTs compared different interventions and concluded that topical corticosteroids were the most effective treatment modality [6]. There are, however, multiple classes and preparations of topical corticosteroids, ranging in cost and efficacy. And not all patients respond favorably to steroids making alternative treatment options necessary.

Despite the large number of potential OLP treatment modalities, few comparisons exist relative to their costs, even at a time when the subject of rising healthcare expenses is a concern. Consequently, we thought an appraisal of OLP treatments relative to reported efficacy and costs might be desirable in helping to guide clinical decision-making and innovative management approaches. The aim of this systemic review was to compare the various topical and systemic therapeutic interventions used for the management of oral lichen planus in terms of their reported efficacy and estimated current costs.

## Materials and methods

To conduct this systematic review, we followed the steps according to the Preferred Reporting Items for Systematic Review and Meta-Analysis (PRISMA).

### Inclusion and exclusion criteria

Included articles were randomized controlled trials (RCTs) that evaluated OLP treatment. RCT eligibility required: (1) studies conducted among adult participants 18 years of age or older; (2) participants with OLP; (3) medication or procedural treatment modalities such as: topical corticosteroids, topical calcineurin inhibitors, systemic therapies, lesion-directed therapy (intra-lesional therapies, phototherapy, laser therapy), natural alternatives, or other topical interventions; (4) measured the treatment efficacy as an outcome, estimated or quantified by various methods of improvement (e.g. different objective and subjective clinical scoring scales/systems). We excluded (1) non-English language papers (2) unavailability of full-text papers; (3) uncontrolled studies without a comparative arm; (4) studies using multiple/combination therapies in single arm, and (5) studies using experimental formulations.

### Search strategy

Systematic literature search was performed from database inception until March 2021 in the electronic databases, MEDLINE via PubMed and the Cochrane library. The search was conducted in PubMed on 03/24/2021 using Medical Subject Heading (MeSH) terms, "Lichen Planus, Oral" and "Lichen Planus, Oral/drug therapy". The search strategy was as follows: ("Lichen Planus, Oral" [Mesh] OR "Lichen Planus, Oral/drug therapy"[Mesh] AND “topical corticosteroids”[Mesh] OR dexamethasone[tiab] OR clobetasol[tiab] OR fluocinonide[tiab] OR triamcinolone[tiab] AND “topical calcineurin inhibitors”[tiab] OR tacrolimus[tiab] OR pimecrolimus[tiab] OR cyclosporine[tiab]) AND (“systemic therapies”[Mesh] OR corticosteroids[tiab] OR hydroxychloroquine[tiab] OR dapsone[tiab] OR azathioprine[tiab] OR “mycophenolate mofetil”[tiab] OR levamisole[tiab] OR retinoids[tiab]) AND (“lesion-directed therapy”[Mesh] OR “intra-lesional steroid injections”[tiab] OR “intralesional BCG-PSN"[tiab]) AND (“phototherapy”[Mesh] OR “photodynamic therapy”[tiab] OR “psoralen and ultraviolet A therapy”[tiab]) AND (“laser therapy”[Mesh]) AND (“topical amelaxanox”[tiab]) OR “topical thalidomide”[tiab] OR “topical retinoids”[tiab]) AND (“natural therapies”[Mesh] OR lycopene[tiab] OR Ignatia[tiab] OR curcumin [tiab] OR “aloe-vera”[tiab]).

### Study selection

Abstracts of the screened articles were reviewed by two authors for eligibility. Any disagreements were judged by a third author. Full text documents of the articles were retrieved and reviewed for final inclusion in the systematic review.

### Data collection and data items

Data extraction was performed independently by eight reviewers. The following information was extracted from each article: author name, publication year, RCT design (single-, double-blind or open-label; parallel or cross-over), treatment modality being studied (strength and preparation, duration, frequency of treatment, treatment outcome and adverse events), sample size (n), therapy assessment (adverse events, relapse rate after successful treatment, follow-up time), cost of therapy and cost of managing the adverse events.

### Risk of bias

For the quality assessment of RCTs, we utilized the Revised Cochrane risk-of-bias tool for randomized trials (RoB2) which involves assessment of six domains: 1. randomization process, 2. assignment to intervention, 3. missing outcome data, 4. measurement of the outcome, 5. selection of the reported result, and 6. overall assessment.

### Outcome measures

The outcome objective and subjective scoring systems utilized by individual studies were considered for assessing the efficacy of different types of treatment modalities employed. The statistical evidence of efficacy between intervention and control was recognized when *p* value < 0.05. Costs of the medications and procedures were retrieved and the range of cost per unit of treatment was calculated using information available on various online pharmacies and websites comparing prescription drug prices with discounted prices (i.e., goodrx.com, singlecare.com, pharmacychecker.com, otc-online-store.com, ebay.com, amazon.com, naturallythinking.com, etc.). The cost was estimated for per unit and per month utilization of the generic or branded equivalents of treatments assessed in RCTs. Costs of the interventions not available in the USA were converted into US dollars; all costs in current dollars.

## Results

### Search results

**Fig. 1:**
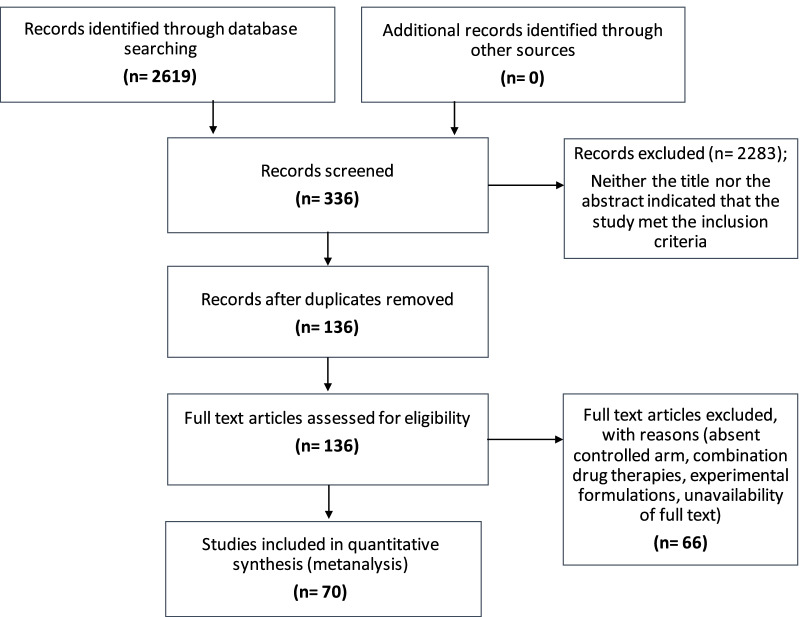
PRISMA flow chart for selection of studies in this systematic review

Two-thousand six hundred nineteen (2619) articles were retrieved using the search strategy. Of these, 70 studies were included in the systematic review*.* Sixty-six full text articles were excluded with reasons {absent controlled arm (35), combination drug therapies (5), experimental formulations (25), unavailability of full text (1)} (Fig. 1).

### Study characteristics

70 studies (total of 2612 patients) published between 1977 and 2020 met the inclusion criteria: Four were single-blinded, three were triple-blinded, six were open-label trials, and the remaining were double-blinded. 67 trials had a parallel RCT design and three had a cross-over design. Eighteen RCTs were placebo-controlled, and the remaining 53 trials compared 2–4 treatment modalities. Key characteristics of included studies are listed in Table 1.

**Table 1:** Key characteristics of the included randomized clinical trials in this systematic review

Topical steroids	Reference Study	Intervention	Comparative agent	No. of pts	Indication	Duration	Frequency	Outcome measure	Results	ADRs	Efficacy Comparison	Level of evidence
Dexamethasone	Bakhtiari [27]	Dex solution 0.5 mg/5 mL	PDT	30; Dex: 15, PDT:15	Bx-proven clinical OLP	2 wks	QID	VAS, Thongprasom clinical score, clinical severity index	No significant difference between the two gps in efficacy index, sign score, symptom score or clinical severity on post-treatment days 15, 30, 60 and 90; Decreases in symptoms statistically significant in both (p-value NS)	PDT: 3 pts-pain from manipulation of the probe tip	Dex = PDT	High risk of bias
	Hambly [28]	Dex solution 0.5 mg/5 mL	Dex solution 0.5 mg/5 mL self-compounded	9; Dex:4, Dex self-compounded: 5; then cross-over	Symptomatic OLP	7 wks	TID	VAS, TSQM-9, photos, self-assessment	TSQM-9 revealed the compounded mouth rinse more favorable than the self-formulation rinse, with a mean improv. in convenience of therapy (22.25%), onset of action (8.48%), and attained symptom relief (4.18%) (p-value NS)	None	Commercial dex > self-formulated dex	High risk of bias
	Mirza [29]	Dex solution 0.5 mg/5 mL	LLLT vs. PDT	45; 15 in each group (dex, LLLT, PDT)	Erosive OLP	4 wks	QID	VAS and clinical score	Significant difference in sign score changes before and after the treatment in the PDT group (*p* = 0.03), LLLT group (*p* = 0.04) and in dex group (*p* = 0.02); statistically significant difference between PDT (*p* = 0.001) and LLLT (*p* = 0.001) against dex group before and after treatment. Mean improv. in pain significantly greater in dex group in comparison with the PDT and LLLT gps (*p* < 0.001). Efficacy index of PDT group improved significantly more than the LLLT (*p* = 0.001) and corticosteroid gps (*p* = 0.001)	None	VAS: Dex > LLLT = PDT;Efficacy: PDT > LLLT = Dex	High risk of bias
Clobetasol	Rödström [33]	Clo oint. 0.05%	TA paste 0.1%	40; 20 in each	Erosive OLP	9 wks	BIDx3wks, QDx 3wks, once every other dayx3 wks	VAS and 4-point clinical score	Clo more effective than TA at 3 wks (*p* < 0.05). No significant difference following 6 and 9 wks of treatment	NS	Clo > TA (at 3 wks); Clo = TA (6 & 9 wks)	Low risk of bias
	Muzio [30]	Clo oint. 0.05%	Clo in analgesic base vs. Clo in denture paste	24; 8 in each	Bx-proven OLP	2 wks	TID	VAS	Clo effective in each group (*p* < 0.05)	candidiasis (number NS)	Clo oint = Clo + analgesic base = Clo + denture paste	Low risk of bias
	Carbone [31]	Clo oint. 0.025%	Clo oint. 0.05%	35; 15 in	Bx- proven symptomatic OLP	8 wks	BID	VAS and clinical score	VAS improved in both (*p* = 0.001); clinical score improved (*p* < 0.05 in both gps). No statistically significant differences b/w gps	None	Clo oint 0.025% = clo oint 0.05%	Low risk of bias
	Kaur [32]	Clo oint. 0.025%	TC oint. 0.1%	40; 20 in each	Bx- proven symptomatic OLP	4 wks	BID	Symptom and clinical grading score	Improv. in both groups. No statistically significant differences b/w gps	None	Clo oint. 0.025% = TC oint. 0.1%	Low risk of bias
	Arduino [8]	Clo gel 0.05%	Placebo	32; 16 in each group	OLP	8 wks	BID	VAS and 4-point clinical score	Clo: reduction in VAS and clinical score in tx (*p* = 0.005)	Clo: 1 pt-GERD; 1pt- mild elevated FBS; placebo: 1pt- skin rxn	Clo > placebo	Low risk of bias
Fluocinonide	Voute [10]	Fluocinonide oint. 0.025%	Placebo	40; 20 in each group	Bx- proven OLP	9 wks	6 × daily	VAS; 4-point clinical score	Statistically significant improv. in fluocinonide group objectively (*p* = 0.0013) and symptoms (*p* = 0.008)	None	Flu > placebo	Low risk of bias
	Carbone [34]	Fluocinonide oint 0.025%	Clo oint. 0.05% vs. placebo	60 (Flu:25, Clo:24, placebo:11)	Atrophic-erosive symptomatic OLP	24 wks	TIDx 8wks; BIDx 8wks; QDx 4 wks	Objective and subjective clinical progress score	Clo more effective in atrophic areas (75% vs 25% of total response, respectively) (*p* = 0.004)	None	Clo > Flu	Low risk of bias
Triamcinolone	Sieg et al. [43]	TA paste 0.1%	Cyclosporin oily liquid preparation	13; CsA:6, TA:7	Bx-proven OLP	6 wks	TID	7-point mucosal lesion scoring	Clinical improv. in both gps, no statistically significant difference between gps (no p-value)	CsA: precipitation of waxy particles during 'swishing' the oily solution; TA: 3 pts- burning	TA = CsA	Some concerns
	Ungrouphaiboon et al. [35]	TA paste 0.1%	TA solution 0.1%	20; TA paste:11, TA rinse:9	Bx-proven symptomatic OLP	4 wks	QID	Clinical response: none, partial (1–33% reduction in lesion), good (34–99% lesion reduction, complete response	No statistically significant difference b/w 2 gps	TA paste group: 2 pts- oral candidiasis	TA paste = TA rinse	Some concerns
	Laeijendecker et al. [38]	TA oint 0.1%	TC oint. 0.1%	40; 20 in each	OLP	6 wks	QID	Reduction in pain	TA: 6 pts healed, 12 showed improv.; TC: 2 pts healed, 7 improved. Initial results better in TC group (*p* = 0.007)	Temporary pain and burning sensation in both gps	TC > TA	Some concerns
	Malhotra et al. [67]	TA paste 0.1%	Oral betamethasone mini pulse (5 mg twice/wk)	49 (TA: 24, BM: 25)	Bx-proven symptomatic OLP	24 wks	TA: TID × 12 wks, BID × 4 wks, QD × 4 wks, alternate days × 4 wks; BM: 5 mg × 12 wks, 4 mg × 4 wks; 3 mg x 4wks; 2 mg × 4 wks	Clinical score (based on number of sites and area affected) and change in symptoms	Clinical score: reduction in severity score more in TA group (*p* = .026); No statistical difference in symptomatic improv. b/w 2 gps	TA group: 5 pts-candidiasis, 1 pt epigastric discomfort; BM group: 7 pts- facial edema, 7 pts epigastric discomfort, 5 pts-fatigue, 4 pts hand/foot edema, 1pt diabetes mellitus	Clinical score: TA > ; Symptoms: TA = BM	High risk of bias
	Mansourian et al. [47]	TA paste 0.1%	AV solution	46; 23 in each	Bx-proven OLP	4 wks	QID	VAS, Thongprasom score, lesion size (grid)	Both AV and TA significantly reduced VAS, Thongprasom score and lesion size (*p* < 0.001). No significant difference b/w 2 gps	None	TA = AV	Low risk of bias
	Handa [37]	TA paste 0.1%	Fluticasone propionate spray 0.05%	40; 20 in each group	Symptomatic OLP	8 wks, 2 wks washout, 8 wks crossover	TA: QID; Fluticasone: 50 μg, 2 dose unit BID	Clinical scoring, VAS, OHIP-14	No statistically significant difference b/w 2 gps (p value NS)	NS	TA = fluticasone spray	Some concerns
	Amanat et al. [54]	TA paste 0.1% in orabase	Cryotherapy (NO)	30 (one side intervention, the other side control)	Bx-proven, bilateral OLP	4 wks	TID	Lesion size, RPAE score	Both treatments reduced the sign scores and severity significantly (*p* < 0.05), no significant differences between gps (*p* > 0.05)	Cryotherapy: 17 pts- minor swelling. 12 pts- pain in first 7–10 days	TA = cryotherapy	High risk of bias
	Kia et al. [48]	TA paste 0.1%	Curcumin paste 5%	50; 25 in each group	Clinical and bx-proven OLP	4 wks	TID	VAS and Thongprasom score	No significant difference between the two gps in VAS (VAS at baseline: *p* = 0.17; VAS two weeks later: *p* = 0.3; VAS four weeks later: *p* = 0.46) or Thongprasom score (baseline: *p* = 0.77, two weeks later: *p* = 0.92, four weeks later: *p* = 0.31)	Curcumin: burning sensation, itching, mild swelling and xerostomia, yellow gingiva; TA: 1 burning and 1 mucosal desquamation	TA = Curcumin	Some concerns
	Sivaraman et al. [36]	TA paste 0.1%	Clo oint. 0.05%, vs. TC oint. 0.03%	30; 10 in each of the 3 gps	Atrophic, ulcerative OLP	6 wks	QID	Reduction in lesion size	TA and Clo: significant reduction in lesion size than Tac gp; overall better results with Clo (*p* = 0.005)	None	Clo > TA > TC	Some concerns
	Thomas et al. [49]	TA paste 0.1%	Curcumin gel 1% TID vs. curcumin gel 6x/d	75; 25 in each of the 3 gps	Bx-proven symptomatic OLP	12 wks	TA: TID; curcumin: TID; 6x/d	Numerical Rating Score (burning) and Modified Oral Mucositis Index	Reduction in burning and erythema/ulceration (*p* < 0.001) in all 3 gps. TA showed max. reduction in burning sensation (77% change) and erythema/ulceration (67% change) (*p* < 0.001)	None	TA > curcumin gel 1% 6x > curcumin gel 1% TID	High risk of bias
	Singh et al. [40]	TA paste 0.1%	Dapsone 100 mg vs. TC oint. 0.1% vs. topical retinoid (type NS)	40; 10 in each of the 4 gps	Reticular, erosive, atrophic, plaque-like OLP	12 wks	BID	Symptoms and signs scored according to Raj et al. and Kaliakatsou et al. scales	All clinical improv. (*p* < 0.05), steroidal and non-steroidal agents had equal efficacy. Of the non-steroidal drugs, oral dapsone had greater efficacy than topical retinoid (*p* < 0.05); no significant differences between oral dapsone and topical tacrolimus (*p* > 0.05) or between topical retinoid and TC (*p* > 0.05)	Mild tingling in the oral cavity in patients treated with topical agents	Dapsone > TA = TC = retinoid	Some concerns
	Siponen et al. [39]	TA paste 0.1%	TC oint. 0.1% vs. placebo	18; TA: 7, TC: 11, placebo: 9	Bx-proven symptomatic OLP	9 wks	TID	VAS and clinical score	Reduction in both TC and TA gps as compared to placebo (*p* = 0.012 and 0.031). No statistically significant difference b/w 2 gps	TA: 3 pts-burning, tingling, gingival tenderness, 2 pts-candidiasis	TA = TC	Low risk of bias
	Li et al. [1]	TA paste 0.1%	S. Salivarius K12 lozenge	40; 20 in each	Symptomatic OLP	4 wks	TA: TID; Lozenges: BID	Sign scores and VAS	No statistical difference was observed between two gps after 4-week treatment in sign scores (*p* = 0.063) or VAS (*p* = 0.698)	None	TA = S. Salivarius K12	High risk of bias
	Bakshi et al. [27]	TA solution 0.1%	Nanocurcumin gel 1%	31; 17 in TA + placebo, 14 in TA + NC	Symptomatic OLP	4 wks	TID	REU score and efficacy index	Both had significant improv. in REU score and efficacy score, TA + NC group significantly better in both measures than TA + placebo (*p* < 0.001)	NS	Nanocurcumin gel > TA	Low risk of bias
Betamethasone	Tyldesley and Harding[11]	BM valerate aerosol (2 puffs/dose); daily dose: 800/ug	Placebo	23; BM: 12, placebo: 11	Symptomatic OLP	8 wks	QID	Lesion size, discomfort/pain	BM: improv. of lesion size and pain in 8 vs. 2 in placebo (*p* < 0.05)	BM: 1 pt-oral candidiasis	BM > placebo	Low risk of bias
Fluocinolone	Thongprasom et al. [7]	Fluocinolone acetonide 0.025% in orabase	TA 0.1% in orabase	40; 20 in each	Bx-proven OLP	4 wks	QID	5-point Thongprasom clinical score	Fl: lesions in 13/19 pts effectively cured, TA: 8/19 pts cured (*p* < 0.05)	Oral candidiasis: Fl- 9 pts; TA-4pts	Fluocinolone > TA	High risk of bias
*Calcineurin inhibitors*												
Tacrolimus	Radfar et al. [55]	TC oint. 0.1%	Clobetasol gel 0.05%	29; TC:15, clo:14	Erosive OLP	6 wks	QID x 2wk; TID X 2wk; BID X1 wk; QHS × 1 wk	Complete resolution of the clinical signs and symptoms	82.6% in tacrolimus and 81.6% in the clobetasol group – improv., (*p* < .0001)	Discomfort, burning and tingling	TC > Clo	Low risk of bias
	Corrocher et al. [56]	TC oint. 0.1%	Clobetasol oint. 0.05%	32; 16 in each	OLP	4 wks	QID	Pain severity, burning sensation, 4-point clinical score	TC group- low median pain score *p* < 0.001; Clo group- low pain score *p* < 0.05 but mild increase in the median severity scores	None	TC > Clo	Low risk of bias
	Sonthalia and Singal [57]	TC oint. 0.1%	Clobetasol oint. 0.05%	40; 20 in each	OLP	8 wks	TID	VAS, Clinical score	VAS and clinical score decreased (*p* < 0.05) in both gps, but no significant diff b/w 2 gps	Burning and increased sensitivity	TC = Clo	Low risk of bias
	Vohra et al. [59]	TC oint. 0.1%	PI cream 1%	40; 20 in each	Erosive, OLP	8 wks	BID	Clinical score	Significant reduction in the clinical severity score in both pimecrolimus and tacrolimus (*p* < 0.05)	None	TC = PI	Low risk of bias
	Hettiarachchi et al. [58]	TC cream 0.1%	Clobetasol cream 0.05%	68; 34 in each	OLP	3 wks	BID	VAS, Thongprasom clinical response	TC: mean pain score dropped by 1.59 (R) and 1.53 (L), clinical score reduced by 1.18 (R) and 1.0 (L); Clo: VAS drop by 0.94(R) and 0.85 (L) & clinical score reduced by 0.5 (R) and 0.26 (L) (*p* < 0.05)	None	TC > Clo	Low risk of bas
Pimecrolimus	Swift et al. [12]	PI cream 1%	Placebo	20; 10 in each	Erosive OLP	4 wks	BID	Lesion size, VAS	PI more effective; VAS decreased (*p* = 0.02)	None	PI > Placebo	Low risk of bias
	Passeron et al. [13]	PI cream 1%	Placebo	12; 6 in each	Erosive OLP	4 wks	BID	12-point clinical score & VAS	PI effective; Mean score 6.83 on day 0 vs. 3.33 on day 28 in PI arm (*p* = 0.04)	PI: 2 pts transient burning sensation	PI > Placebo	Low risk of bias
	Gorouhi et al. [41]	PI cream 1%	TA cream 0.1%	40; 20 in each	OLp > 8 yrs	8 wks	QID	VAS, OHIP score & objective clinical score	No significant difference b/w 2 arms in VAS (*p* = 0.70), OHIP (*p* = 0.38), clinical score (*p* = 0.86)	PI: 2pts- transient burning; TA: none	PI = TA	Low risk of bias
	Volz et al. [14]	PI cream 1%	Placebo	20; 10 in each	Erosive OLP	4 wks	BID	Composite score (mucosal erosions and pain sensation)	Composite score reduced in PI arm (*p* = 0.025)	PI: 4 pts-burning sensation, 1 pt- mucosal paresthesia; Placebo:1 pt- mucosal paresthesia	PI > Placebo	Low risk of bias
	McCaughey et al. [15]	PI cream 1%	Placebo	21; PI: 10, placebo: 11	Erosive OLP	6 wks	BID	Investigator’s Global Assessment of severity, pain, erosion size	PI superior in reducing mean pain and erosion size (mean size 11.10 at baseline vs. 3.70 at week 6) (*p* = 0.02)	None	PI > Placebo	Low risk of bias
	Arduino et al. [9]	PI cream 1%	TC oint. 0.1%	30; 15 in each	Topical steroid refractory OLP	8 wks	BID	Symptomatic improv., therapeutic effectiveness	Both effective; no statistically significant difference b/w 2 arms	PI: 2pts- xerostomia, 2pts-GERD, 1pt-herpes labialis; TC: 2pts burning,	PI = TC	Low risk of bias
	Arunkumar et al. [46]	PI cream 1%	TA paste 0.1%	30; 15 in each	Bx-proven symptomatic OLP	8 wks	QID	VAS, mean clinical score and erythematous area	Reduced clinical score in PI arm (*p* < 0.01); no statistically significant diff in reduction of VAS (*p* = 0.18) & erythema (*p* = 0.07)	None	Clinical score: PI > TA; VAS: PI = TA	Low risk of bias
	Pakfetrat et al. [42]	PI cream 1%	TA cream 0.1%	28; 14 in each	Atrophic-erosive OLP	8 wks	TID	Thongprasom lesion scoring, VAS	Both effective; No statistically significant difference	None	PI = TA	Low risk of bias
	Ezzatt and Helmy [60]	PI cream 1%	Betamethasone valerate cream 0.1%	30; 15 in each	Atrophic-erosive OLP	4 wks	QID	Clinical score, VAS	Both showed reduction in clinical score and VAS (*p* < 0.001) but no statistically significant diff b/w 2 arms in 4 wks; PI: 33% clinical score reduction, 57.5% VAS reduction; BM:13.9% clinical score reduction and 30.6% VAS reduction after 1 wk	PI: 4 pts-burning,2 pts-dysguesia; BM: 2pts-burning, 1pt-dysguesia	PI = BM	Low risk of bias
Cyclosporine	Eisen et al. [16]	CsA solution 100 mg/ml	Placebo	16; 8 in each	Bx-proven symptomatic OLP	8 wks	TID	Pain (4-grade scale), erosion (4-grade scale)	CsA: improv. in erythema (*p* = 0.003), erosion (*p* = 0.02), reticulation (*p* = 0.007), pain (*p* = 0.002)	CsA: transient burning on swishing in all pts	CsA > placebo	Low risk of bias
	Harpenau et al. [17]	CsA solution 100 mg/ml	Placebo	14; 7 in each	Bx-proven erosive OLP	4 wks	QD	VAS; lesion character (ulcer, erythema & reticulation) & size	CsA: significant reduction in erythema, ulceration, and VAS; p-value NS	None	CsA > placebo	Low risk of bias
	Lopez [61]	CsA solution 1%	TA solution 0.1%	20; 10 in each	Bx proven OLP	8 wks	TID	Symptom, erosion and erythema score	CsA: greater decrease of symptoms (90% vs. 60% in TA), erythema and erosion; p-value NS	NS	CsA > TA	Low risk of bias
	Femiano et al. [63]	CsA solution 100 mg/ml	IM sul 600 IU, then oral doses 250 IU	20; 10 in each	Topical steroid recalcitrant bx-proven OLP	4 wks	CsA: TID, Sul:BID	Pain relief, clinical resolution of erosion/ulceration	Sulodexide more effective- clinical resolution faster than CsA at a mean of 36 days and pain resolution in 90% by mean 6.4 days (*p* < 0.004)	CsA: None; Sul: vertigo, vomiting and hot flushes	Sul > CsA	High risk of bias
	Yoke et al. [44]	CsA solution 100 mg/ml	TA paste 0.1%	139; CY: 68; TA:71	Bx proven OLP	8 wks	TID	VAS; Thongprasom clinical grading	No statistically significant difference b/w two arms	TA: 3 pts- transient burning; CsA: 14 pts- burning; 4 pts- GI upset; 1pt- lip swelling & itching	CsA = TA	Low risk of bias
	Thongprasom et al. [45]	CsA solution 100 mg/ml	TA paste 0.1%	13; CsA:6, TA:7	Bx proven symptomatic OLP	8 wks	TID	VAS, Thongprasom clinical grading (5-point)	No statistically significant differences b/w 2 gps	CsA: 5 pts- burning sensation, itching, swelling lips, petechial hemorrhage; TA: None	CsA = TA	Low risk of bias
	Georgaki et al. [62]	CsA solution 100 mg/ml	Dex rinse 0.5 mg/5 ml	32; 16 in each	Bx proven symptomatic OLP	4 wks	TID	VAS; Thongprasom clinical grading, dysphagia and speech difficulties	Dex: better in clinical scoring (*p* = 0.001). No significant diff b/w 2 gps in improv. of pain, dysphagia and speech difficulties	NS	ClinicaL score: Dex > CsA; VAS: Dex = CsA	Low risk of bias
*Other topical agents*												
Amlexanox	Verma [52]	AX paste 5%	TA paste 0.1%	60; 30 in each	Symptomatic reticular/erosive OLP	12 wks	QID	VAS; clinical sign stage: erythematous areas, white striae + lesion size	TA more effective > AX. AX: 60% reduction in the clinical sign stage & TA: 98% reduction (*p* < 0.05); VAS = no significant difference	None	Clinical score: TA > AX; VAS: TA = AX	Low risk of bias
Retinoid	Giustina et al. [18]	isotretinoin gel 0.1%	Placebo	22;11 in each	Ulcerated lichen planus	8 wks	BID	Reduction in pain and erythema-severity scale (0–5)	Significant improv. in topical retinoid group with statistically significant (*p* < .002); Reduction in severity scale 3.0 to 1.7 after 8 weeks	Burning and superficial desquamation	Isoretinoin > Placebo	Low risk of bias
	Petruzzi et al. [20]	Tazarotene cream 0.1%	Placebo	12; 6 in each	Hyperkeratotic OLP	8 wks	BID	6-degree score scale, reduction in lesion	4 patients healed, 2 patients improved in tazarotene and 5 patients with no improv. and 1 worsening (*p* = 0.0049)	Burning, taste abnormalities	Tazarotene > Placebo	Low risk of bias
	Piattelli et al. [19]	Isotretinoin gel 0.1%	Placebo	20; 10 in each	Bx proven OLP	16 wks	TID	Complete healing of the lesions	Isoretinoin: 60% complete healing (*p* = 0.029)	NA	Isoretinoin > Placebo	Low risk of bias
Tocopherol	Bacci et al. [21]	Tocopherol acetate (gelly formulation)	Placebo	33; Tocopherol = 17, Placebo = 16; then cross-over	Bx-proven reticular OLP	4 wks, 2 wk washout, 4 wks crossover	TID	VAS, length of striae, surface area of lesion, Thongprasom score	Significant difference in surface area of lesion (*p* = 0.0045) and Thongprasom score (*p* = 0.0052) in tocopherol group	None	Tocopherol > Placebo	Low risk of bias
*Intralesional*												
Triamcinolone	Ahuja et al. [65]	Intralesional triamcinolone (10 mg/ml)	PRP 0.5 ml	20; 10 in each	Erosive OLP	8 wks	Weekly injection—for 2 to 4 months	VAS, reduction in erythema and size of the lesions	Statistically significant reduction in both gps (*p* < 0.005); no significant difference b/w 2 gps	Intralesional TA: Erythema in 1 pt; PRP: increased VAS score in 1 pt	TA = PRP	Low risk of bias
BCG-PSN	Xiong et al. [64]	Bacillus Calmette–Guerin polysaccharide nucleic acid (BCG‐PSN)	Intralesional triamcinolone (10 mg/ml)	56; BCG-PSN = 31 & TA = 25	Bx-proven erosive OLP	2 wks	BCG-PSN: every other day. TA: every week	VAS & measured erosive areas	No statistical differences b/w 2 gps in erosive areas (*p* = 0.801) and VAS scores (*p* = 0.946)	Burning/swelling at injection site in 9.7% of BCG-PSN group and 8% in TA group	BCG = TA	Low risk of bias
*Systemic Therapies*												
Systemic retinoids	Hersle et al. [22]	Etretinate 25 mg	Placebo	28; 14 in each	Bx-proven OLP for atleast 6 mths	8 wks	TID	4-point clinical scoring	Etretinate: 93% improv. vs. 5% in placebo (*p* < 0.001)	Etretinate: all pts- skin and mucosa dryness; 6 pts-keratoconjunctivitis, rash, headache, itchiness & hair loss	Etretinate > Placebo	Some concerns
Levamisole	Lin et al. [66]	Levamisole 50 mg	(Levamisole + vit B12) and (Vit B12 only)	147; 100 in L + B12 gp, 37 in L gp, & 10 in B12 gp	OLP	2–50 months (mean = 14)	BID if 30–50 kg or TID if 50–70 kg, for 3 days at 2 wk interval	Size & distribution of lesions, pain & burning symptoms	L only group & L + B12 group: 100% objective & subjective improv.; Vit B12 alone: 13% improv. in symptoms and 20% improv. in signs (p-value NS)	None	Levamisole = Levamisole + B12 > B12 only	High risk of bias
*Natural alternative*												
Lycopene (systemic)	Saawaran et al. [23]	Lycopene 4 mg	Placebo	30; 15 in each	Bx proven symptomatic OLP	8 wks	BID	VAS; Tel Aviv-San Francisco scale	Lycopene: 84% VAS reduction, 100% showed > 50% benefit; Placebo: 67% VAS reduction, 66.6% showed > 50% benefit (*p* < 0.05)	None	Lycopene > Placebo	Low risk of bias
Ignatia (topical)	Mousavi et al. [24]	Ignatia 30C liquid	Placebo	30; 15 in each	Bx proven atrophic/erosive OLP	12 wks	QD	VAS and mean lesion size (cm)	Ignatia more effective; Ignatia: mean lesion size- 2.2 cm, VAS-13 mm; Placebo: mean lesion size-4 cm, VAS-40 mm (*p* < 0.05)	None	Ignatia > Placebo	Low risk of bias
Aloe Vera (topical)	Choonhakarn et al. [25]	AV gel 70%	Placebo	54; 27 in each	Bx proven OLP	8 wks	BID	VAS and Thongprasom clinical scale	AV: improved clinical response in 88% and improved burning in 33% vs. 4% in placebo group (*p* < 0.001)	None	AV > Placebo	Low risk of bias
	Salazar-Sánchez et al. [26]	AV gel 70%- 0.4 ml/dose	Placebo	64; 32 in each	Bx proven OLP	12 wks	TID	VAS, Thongprasom clinical scale, OHIP-49	No statistically significant diff in VAS and clinical score at 12 wk; AV showed improv. in total OHIP score (*p* = 0.046)	None	AV = Placebo	Low risk of bias
	Reddy et al. [51]	AV gel 70%	TA 0.1% paste	40; 20 in each	Erosive & atrophic OLP	8 wks	TID	VAS & clinical score	AV: clinical score and VAS significantly better than TA (*p* < 0.05)	None	AV > TA	Low risk of bias
*Other procedural modalities*												
LLLT	Jajarm et al. [68]	Low intensity laser therapy (LILT) 630 nm diode laser	Dexamethasone solution 0.5 mg/5 ml	30 (one side intervention, the other side control)	Erosive-atrophic OLP	4 wks	LILT: BID; Dex: QID	Thongprasom clinical scale, VAS, RAE	Appearance score, pain score, and lesion severity was reduced in both gps (p value NS). No significant differences b/w the treatment gps regarding the response rate and relapse	None	LLLT = Dex	Some concerns
Laser	Agha-Hosseini et al. [72]	CO2 laser irradiation	low-level laser therapy (LLLT)	28 (one side intervention, the other side control)	Oral lichen planus	2 wks	CO2 laser: 1 session; LLLT: 5 sessions	Thongprasom clinical scale, VAS, size of lesions	Lesion size reduction significantly higher in LLLT compared to CO2 (*p* < 0.05). Improv. in clinical signs significantly higher in LLLT (*p* < 0.05). Symptom reduction was significantly higher in LLLT group (*p* < 0.05)	NS	LLLT > CO2 laser	High risk of bias
LLLT	Dillenburg et al. [70]	Laser phototherapy (LPT) 660 nm diode laser	Clobetasol gel 0.05%	42 (one side intervention, the other side control)	Atrophic/erosive OLP	4 wks	LPT- 3x/wk; Clo: TID	Clinical, symptoms, and functional scores	The LPT group had significantly lower clinical scores compared to clobetasol group (*p* = 0.001). Symptom score was maintained at a stable level for the LPT group in the follow up period, whereas a significant increase was found in the clobetasol group (*p* = 0.05)	Clo: 3 pts- Transient burning sensation; LPT: None	LPT > Clo	Low risk of bias
PDT	Jajarm et al. [68]	Toluidine blue for 10 min followed by photodynamic therapy	Dexamethasone rinse 0.5 mg/5 ml	25 (one side intervention, the other side control)	Erosive/atrophic OLP	4 wks	PDT:2x/wk; Dex:QID	Thongprasom clinical scale, efficacy indices, and experienced pain	Statistically significant reduction in sign score for the experimental (*p* = 0.021) and control (*p* = 0.002) gps. Efficacy index of the control group improved significantly more than the experimental group (*p* = 0.001)	None	Dex > PDT	High risk of bias
Laser	Kazancioglu (2015)	A diode laser 808	Ozone vs. dex rinse vs. placebo	120; 30 in each gp	atrophic-erosive OLP	4 wks	Laser:2x/wk; Ozone:2x/wk; Dex: QID	Thongprasom clinical scale, VAS, RAE score	Improv. in all gps but significantly better in Ozone and steroid gps (*p* < 0.05) as compared to laser and placebo	None	Ozone = Dex > Laser > placebo	Some concerns
Laser	Othman et al. [74]	A diode laser 970	TA 0.1% orabase	24 (one side intervention, the other side control)	Erosive-atrophic Reticular	4–5 wks	Laser: 2x/wk; TA: QID	Thongprasom clinical scale, RAE score, TNF α level	TA group showed statistically significantly lower mean RAE score than Laser group (*p* = 0.02) as well as lower TNF-α level	None	TA > laser	Some concerns
Laser	El Shenawy et al. [75]	A diode laser 970	TA 0.1% orabase	24; 12 in each	Erosive-atrophic	Laser: 8 wks; TA: 4 wks	Laser: 2x/wk; TA: QID	VAS, RAE score	Significant improv. in TA group than laser group (*p* < 0.05)	NS	TA > laser	Some concerns
PDT	Lavaee and Shadmanpour [69]	660-nm diode laser for 10 min	Topical TA 0.1%	8 (one side intervention, the other side control)	Atrophic/erosive OLP	PDT: 3 wks; TA: 4 wks	PDT: 1x/wk; TA: TID	Thongprasom clinical scale, VAS, size of lesions	Significant difference in all scores between session 0 and 4 in both gps (*p* < 0.05). Changes in scores between the intervention and comparative gps were not statistically significant (*p* = 0.340)	None	PDT = TA	Low risk of bias
LLLT	Ferri et al. [71]	Clo gel 0.05%	Photobiomodulation (PBM)	34; 17 in each group	Reticular,atrophic, and erosive OLP	4 wks	Clo: TID; PBM: 2x/wk	VAS; Thongprasom clinical score	Decreased pain in both; clinical resolution: clo- 79.4%, PBM- 64.7% (*p* < 0.05)	None	Clo > PBM	Low risk of bias

AV: aloe-vera, BM: betamethasone, Bx: biopsy, b/w: between, BCG‐PSN: Bacillus Calmette–Guerin polysaccharide nucleic acid, Clo: clobetasol, CsA: cyclosporine, Dex: dexamethasone, Flu: fluocinonide, FBS: fasting blood sugar, Gp: group, Improv.: improvement, LLLT: low level laser therapy, LPT: laser phototherapy, Mins: minutes, NS: not stated, NC: nanocurcumin, OLP: oral lichen planus, OHIP: Oral Health Impact Profile, Oint.: ointment, PDT: photodynamic therapy, PBM: photobiomodulation, PI: pimecrolimus, Pt: patient, RAE: reticulation, atrophy, erosion score; RPAE: reticular, white plaque, atrophy, erosion and ulceration clinical score, Rxn: reaction, TA: triamcinolone, TC: tacrolimus, TSQM: Treatment Satisfaction Questionnaire for Medication, Tx: treatment, VAS: visual analog scale, wk: week

### Treatment modalities

The treatment modalities investigated in eligible studies included: topical therapies {dexamethasone (n = 3), clobetasol (n = 6), fluocinonide (n = 2), triamcinolone (n = 14), betamethasone (1), fluocinolone (1), tacrolimus (5), pimecrolimus (9), cyclosporine (7), amlexanox (1), retinoids (3), tocopherol (1)}; systemic therapies {retinoids (1), levamisole (1)}; intra-lesional therapies {triamcinolone (1), Bacillus Calmette-Guerin polysaccharide nucleic acid (1)}; natural alternatives {aloe-vera (3), Ignatia (1), lycopene (1)}; laser (6) and photodynamic therapy (2).

### Outcome measures

For assessing the subjective treatment response, the majority of RCTs (57%) used a visual analog scale (VAS) [7–10, 12, 13, 17, 21, 23–31, 33, 37, 39, 41, 42, 45–48, 51, 53, 57, 58, 60, 62, 64, 65, 68, 69, 71, 73, 75, 76]. While there was significant heterogeneity in the clinical scoring scales used to measure treatment response among studies, the Thongprasom scoring system was used most often (19 RCTs; 27%) [7, 21, 25–27, 42, 44, 45, 47, 48, 58, 62, 68, 69, 71–74, 76]. Alternatively, other scales included the Modified Oral Mucositis Index, the Tel Aviv-San Francisco scale, RAE score (reticulation, atrophy, erosion), RPAE score (reticular, white plaque, atrophy, erosion and ulceration), and the REU (reticulation, erosion, ulceration) score [23, 49, 50, 54, 73–75].

### Efficacy (objective and subjective improvement)

The two primary efficacy endpoints reported in the RCTs were objective improvement (reduction in the clinical score or severity) and subjective improvement (reduction in pain/VAS). Most studies (57%) showed statistically significant results (*p* < 0.05) supporting the effectiveness of their respective interventions. Based on the RCTs results, we created a consensus list reflecting the level of efficacy from most efficacious to the least for steroidal and non-steroidal modalities (Additional file 1: Table S1).

#### Placebo-controlled trials (18)

Of the 70 trials, 18 compared an intervention to placebo. The following were associated with statistically significant improvements in pain and lesion response compared to placebo: clobetasol gel 0.05% [8], fluocinonide ointment 0.025% [10], betamethasone valerate aerosol [11], pimecrolimus cream 1% [12–15], cyclosporine solution 100 mg/ml [16, 17], isoretinoin gel 0.1% [18, 19], tazarotene cream 0.1% [20], tocopherol gel [21], systemic retinoid [22] and the three natural alternatives (oral lycopene 4 mg, Ignatia 30 C liquid and aloe-vera gel 70% [23–25]. There was a single placebo-controlled trial (n = 4) comparing aloe-vera gel 70% with placebo that did not demonstrate statistically significant superiority of the intervention [26].

#### RCTs comparing interventions

##### Topical Dexamethasone (Dex)

Commercially available dexamethasone solutions 0.5 mg/5 ml were associated with better clinical outcomes than self-compounded dex [27]. One study comparing dex to photodynamic therapy (PDT) found no difference in efficacy [28], while another comparing dexamethasone, PDT, and low-level laser therapy (LLLT) found dex to be most effective in reducing the pain score and PDT to be most effective in improving the clinical lesions [29].

##### Topical Clobetasol (Clo)

Studies comparing delivery methods of clobetasol 0.05%- clo ointment vs. clo in oral analgesic base vs. clo in denture paste (n = 24) and concentrations of clo (0.025% vs. 0.05%) found each to be effective in reducing pain with additional improvement in clinical scores in the latter (n = 35) [30, 31]. Clo ointment 0.025% was also shown to be comparable to tacrolimus ointment 0.1% (n = 40) [32].

In comparison to triamcinolone paste 0.1%, clo ointment 0.05% showed greater efficacy at 3 weeks of treatment, however, at 6 and 9 weeks of treatment, there was no significant difference between the two (n = 40) [33]. Clo ointment 0.05% demonstrated greater efficacy in reducing objective scores than fluocinonide ointment 0.05% and placebo (n = 60) [34].

##### Topical Triamcinolone (TA)

Over a third of the RCTs (26/70; 37%) studied the efficacy of TA paste 0.1%. The two formulations of TA paste and TA solution were determined to be equally efficacious [35]. Three RCTs (n = 30, 40 and 40) comparing TA paste 0.1% with other topical steroids found that clobetasol 0.05% ointment and fluocinolone acetonide 0.025% in orabase were more efficacious than TA [7, 36] but fluticasone spray 0.05% was equally efficacious to TA [37].

In comparison to tacrolimus (TC) ointment, four RCTs (n = 40, 30, 18 and 40) found different results, with TA paste 0.1% shown to be inferior to TC ointment 0.1% [38], superior to TC ointment 0.03% [36] and equal to TC ointment 0.1% [39, 40] in terms of clinical improvement. Two RCTs (n = 40 and 28) comparing pimecrolimus cream 1% with TA cream 0.1% [41, 42], and three RCTs (n = 13, 139 and 13) comparing cyclosporine solution with TA paste 0.1% found no statistically significant difference between these therapies [43–45]. A double-blind RCT (n = 30) comparing pimecrolimus cream 1% with TA paste 0.1% showed a mixed outcome, with TA showing equal efficacy in reducing VAS but reduced efficacy in reducing the clinical score at 8 weeks of treatment [46].

In comparison to natural alternatives, the results were mixed. While two RCTs (n = 46 and 50) found TA paste 0.1% to be equally efficacious to aloe-vera (AV) solution and curcumin paste 5% respectively [47, 48]; one study (n = 75) showed that TA paste 0.1% was better than curcumin gel 1% [49] and another study (n = 31) showed nanocurcumin gel 1% was better than TA solution [50]. A double blind RCT (n = 40) comparing AV gel 70% to TA paste 0.1% for 8 weeks showed that OLP clinical score and VAS was statistically significantly better in the AV arm [51].

A trial (n = 60) showed that TA paste 0.1% was more effective than amlexanox paste 5% (anti-inflammatory agent) in improving clinical signs but there was statistically insignificant different between the two in terms of reduction of VAS [52]. No statistically significant difference was observed between TA paste 0.1% vs. *S. salivarius* K12 probiotic lozenge (n = 30) [53] or between TA paste 0.1% and cryotherapy with nitrous oxide (n = 40) with respect to VAS and objective scores [54].

##### Topical Tacrolimus (TC)

Four trials compared different topical formulations of clobetasol and TC. Two trials (n = 29 and 32), showed TC ointment 0.1% was superior to clobetasol gel 0.05% and clobetasol ointment 0.05%, respectively [55, 56]; however, the third RCT (n = 40) demonstrated no significant difference between TC ointment 0.1% and clobetasol ointment 0.05% [57]. The fourth RCT compared TC cream 0.1% (compounded) and clobetasol cream 0.05% (n = 68) and found TC cream to be more effective in reducing VAS and clinical response score [58].

##### Topical Pimecrolimus (PI)

Two RCTs (n = 40 and 30) compared PI cream 1% and tacrolimus ointment 0.1% and showed no statistically significant difference between the two in therapeutic effectiveness [9, 59]. Additionally, the efficacy of PI cream 1% was found to be equal to betamethasone valerate cream 0.1% in reducing clinical score and VAS (n = 30) [60].

##### Topical Cyclosporine (CsA)

When CsA solution 100 mg/ml (with a 10% dilution in olive oil) was compared with triamcinolone solution 0.1% (n = 20), there was greater symptomatic and clinical improvement in the CsA group after 8 weeks, although, p-value was not stated [61]. On the other hand, dexamethasone solution 0.5 mg/5 ml was found to be significantly better than CsA solution 100 mg/ml (n = 32) in reducing the clinical score (although both were equally effective in improving VAS) [62].

An open-label trial (n = 20) comparing sulodexide, a systemic heparinoid, with topical CsA (100 mg/ml solution) showed that sulodexide (one dose of I/M followed by oral doses) led to a faster clinical resolution [63].

##### Intralesional therapies

The two RCTs included in this systematic review that evaluated intralesional therapies compared intralesional triamcinolone (TA) 10 mg/ml with Bacillus Calmette-Guérin polysaccharide nucleic acid (BCG-PSN) and autologous platelet rich plasma (PRP). Intralesional injection of the immunomodulatory extract of BCG administered every other day was found to be equally effective as weekly administration of intralesional TA (n = 56) in reducing lesion size and VAS in OLP [64]. Similarly, the RCT comparing intralesional TA and PRP (n = 20) did not find any significant difference between the two arms [65].

##### Systemic therapies

An anti-helminthic and immunomodulatory agent, levamisole (not available in US), was studied in a triple arm open label RCT (n = 147) comparing levamisole 50 mg vs. vitamin B12 vs. combination of levamisole + B12 [66]. The results showed clinical and symptomatic improvement in all patients in both the levamisole arm and the levamisole + vitamin B12 arm, but the p-value was not-stated.

Dapsone, another immunomodulatory agent, showed the highest clinical and symptomatic improvement in a four-arm open-label RCT (n = 40) comparing oral dapsone 100 mg vs. TA paste 0.1% vs. TC ointment 0.1% vs. topical retinoid (type not stated in the study) after 12 weeks [40]. Another open-label trial (n = 49) comparing TA paste 0.1% with systemic betamethasone (mini-pulse therapy with oral betamethasone 5 mg on 2 consecutive days/week) for 24 weeks, found significant reduction in clinical severity score in the TA group but no difference in the symptomatic improvement between the two groups [67].

#### Laser and Photodynamic therapies

Eleven RCTs studying laser and photodynamic therapies (PDT) met the inclusion criteria. When comparing PDT with topical steroids, the studies indicated mixed results- one study (n = 45) showed superiority of PDT over dexamethasone [29], another (n = 25) showed inferiority to dexamethasone [68], and two studies (n = 30 and 8) showed equal efficacy (PDT = dex and PDT = triamcinolone paste 0.1%) [27, 69]. Similar mixed results were seen with LLLT, and topical steroids- one study (n = 42) showed increased efficacy (LLLT > clobetasol gel 0.05%), another (n = 34) showed reduced efficacy (clobetasol gel 0.05% > LLLT) and the third (n = 30) showed equal efficacy (LLLT = dexamethasone) [70–72].

Dexamethasone solution and triamcinolone paste 0.1% showed higher efficacy than laser therapies (n = 120, 24 and 24) [73–75]. In comparing the clinical efficacy of the three phototherapies, a direct comparison trial (n = 45) showed PDT to be more efficacious than LLLT [29] and the second (n = 28) showed superior results with LLLT than carbon dioxide laser [76].

### Adverse reactions

Twenty-six studies reported adverse drug reactions (ADRs) (Additional file 2: Table S2). Most topical interventions were associated with mild, local ADRs. Oral candidiasis was a common documented ADR of topical corticosteroids (clobetasol, triamcinolone, betamethasone and fluocinolone) [7, 11, 30, 35, 67]. Oral burning sensation was associated with topical agents- tacrolimus, pimecrolimus, cyclosporine, triamcinolone, retinoids, and curcumin [9, 13, 14, 16, 18, 20, 38–41, 43–45, 48, 55, 57, 60, 68]. Overall, topical regimens were well-tolerated without evidence of systemic ADRs.

While patients treated with systemic therapies such as levamisole and lycopene did not experience any local or systemic side-effects, significant systemic side effects including skin dryness, keratoconjunctivitis, rash, headache, itchiness, and hair loss were reported in patients treated with etretinate, a systemic retinoid [22]. ADRs such as vertigo, vomiting and hot flushes were documented in patients treated with sulodexide [63]. Intralesional therapies were associated with local erythema (TA), increased pain (PRP) and burning/swelling at injection site (BCG-PSN and TA) in a subset of patients [64, 65].

Among patients treated with cryotherapy using nitrous oxide, the majority experienced local swelling at the treatment side [54]. None of the studies reported any side effects associated with laser therapy; only one study on PDT reported pain upon manipulation with probe tip [27].

### Assessment of risk of bias

At the individual study level, most of the domains were with low risk of bias. The overall assessment of the risk of bias showed that 49 (70%) studies had low risk of bias, 11 (15.7%) studies had high risk of bias, and 10 (14.2%) studies had some concern.

### Cost of therapeutics

**Fig. 2:**
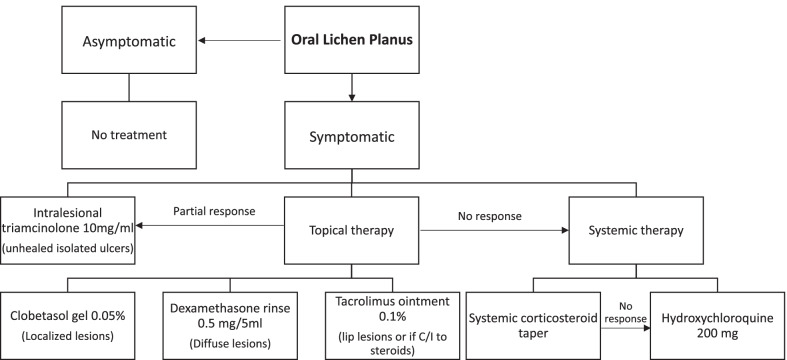
Treatment recommendations for treatment of OLP based on expert opinion

Table 2 presents the estimated costs (U.S. dollars) for the studied interventions. The costs range of topical steroids and topical calcineurin is from $0.04–14.13/unit and $1.13–10.16/unit respectively. The cost of commonly used and commercially available topical therapies is as follows (from highest to lowest): cyclosporine solution > pimecrolimus cream > tacrolimus ointment > clobetasol gel > clobetasol ointment > dexamethasone solution > fluocinonide ointment > betamethasone cream > triamcinolone paste. The cost of intralesional triamcinolone (10 mg/ml) ranges from $10.24–17.00 per ml, but this excludes the procedural cost. Among the systemic medications, the cost of betamethasone was the lowest and oral dapsone was the highest. Considering the costs of different therapeutics and their efficacies, treatment recommendations for OLP have been made based on expert opinion (Fig. 2).

**Table 2:** Estimated cost per unit and per month of common commercially available oral lichen planus interventions studied in the included randomized controlled trials

Intervention	Estimated cost per unit*	Estimated cost per month**
*Topical steroids*		
1. Dexamethasone solution	$0.04–0.27 per ml	$77.50
2. Clobetasol ointment	$0.44–7.8 per g	$123.60
3. Clobetasol gel	$0.76–8.33 per g	$136.35
4. Fluocinonide ointment	$0.39–2.68 per g	$46.05
5. Fluocinonide gel	$1.19–3.45 per g	$69.60
6. Triamcinolone ointment	$0.17–0.44 per g	$9.15
7. Triamcinolone paste	$0.30–1.06 per g	$20.40
8. Betamethasone valerate cream	$0.43–0.96 per g	$20.85
9. Fluocinolone acetonide ointment	$0.52–2.91 per g	$51.45
*Topical calcineurin inhibitors*		
1. Tacrolimus ointment	$1.26–8.66 per g	$148.80
2. Tacrolimus cream	$1.13–3.39 per g	$67.80
3. Pimecrolimus cream	$2.06–10.16 per g	$183.30
4. Cyclosporine rinse	$1.83–8.66 per ml	$2622.50
*Other topical agents*		
1. Amlexanox paste (not available in US)	$0.28–1.38 per g	$24.90
2. Isotretinoin gel	$0.63–1.34 per g	$29.55
3. Tazarotene cream	$1.28–3.66 per g	$74.10
4. Tocopherol acetate gel	$0.15–0.27 per g	$6.30
5. Ignatia liquid	$0.14–0.64 per ml	$195.00
6. Aloe vera gel	$0.02–0.06 per g	$1.20
7. Curcumin gel	$1.48–3.18 per g	$69.9
8. Nanocurcumin gel	$0.27–0.45 per g	$10.8
9. S. salivarius K12 lozenge	$0.59–1.13 per lozenge	$51.60
*Intralesional therapies*		
1. Triamcinolone	$10.24–17.00 per ml	$54.48
2. BCG-PSN	$2.67–3.26 per ml	$44.48
*Systemic therapies*		
1. Lycopene	$0.07–0.19 per capsule (4 mg)	$7.80
2. Oral dapsone	$0.73–3.12 per tablet (100 mg)	$115.50
3. Oral betamethasone	$0.52–0.64 per tablet (0.5 mg)	$46.60
*Other procedure-directed therapies*		
1. Photodynamic therapy	$100–4000 per treatment	$16,400.00
2. Low level laser therapy	$30–200 per treatment	$920.00
3. CO2 laser	$450–1450 per treatment	$7600

*Based on information available on websites- goodrx.com, singlecare.com, pharmacychecker.com, otc-online-store.com, rupills.com, usaherbalmart.com, ebay.com, amazon.com, adooq.com, sastasundar.com, naturallythinking.com, aaos.org, plasticsurgery.org

**Calculated based on mean price per unit and the following amounts dispensed: 500 mL for solutions; 30 g for ointments, gels, and creams; BID for lozenges; 1 mL weekly for intralesional triamcinolone; 1 mL every other day for intralesional BCG-PSN; BID for lycopene and dapsone; 5 mg twice weekly for betamethasone; twice weekly photodynamic therapy, low level laser therapy, and CO2 laser sessions

## Discussion

Ideal therapies are cost-effective, efficacious, and carry a low risk of local or systemic toxicity. The preferred modality for treating OLP is topical therapy due to ease of application, liberty to modify the frequency and duration of treatment and lack of systemic side-effects [5]. Important considerations in choosing a topical regimen include the location, extent of the lesions, and patient tolerability. Gels, ointments, and pastes are best used for focal lesions. For lesions that are more diffuse and/or difficult to access, solutions are preferable, though adequate contact time (3–5 min) must be ensured.

Consistent with other reviews, we found that OLP responds to a wide range of topically delivered medications and procedures including topical steroids (dexamethasone, clobetasol, fluocinonide, triamcinolone), topical calcineurin inhibitors (tacrolimus, pimecrolimus, cyclosporine), topical retinoids, intra-lesional triamcinolone, aloe-vera gel, photodynamic therapy and low-level laser therapies in OLP management.

Comparatively, the high potency topical steroid, clobetasol with an average cost of ~ $4.12/g for the ointment formulation and $4.54/g for the gel formulation, was found to be efficacious compared to topical fluocinonide, triamcinolone and tacrolimus [34, 36]. Contrastingly, three RCTs demonstrated higher efficacy of topical tacrolimus over topical clobetasol, with the average cost of tacrolimus being about $4.96/g [55, 56, 58]. Triamcinolone paste 0.1%, a low potency steroid, costs the least (average cost $0.68/g) among the topical steroids and calcineurin inhibitors. Topical pimecrolimus was comparable to topical triamcinolone, topical betamethasone, and topical tacrolimus [9, 42, 60], but the average cost of pimecrolimus ($6.11/g) was comparatively higher. The higher cost of topical calcineurin inhibitors discourages their use as first-line therapy in OLP management.

Intralesional steroid therapy has been shown to be efficacious but can be deemed invasive, technique sensitive with need for repeated procedures [64, 65]. While the average cost of triamcinolone solution (10 mg/ml) is roughly $13.62/ml, the total cost would also include the procedural cost of the injection itself. Although PDT and laser therapy were shown to be efficacious lesion-directed therapies without significant side-effects [70, 72, 76], the range of cost per treatment session was highest among all the treatment modalities. Among natural alternatives, aloe-vera gel was shown to be comparable to triamcinolone paste 0.1% [51], with the most modest price of $0.04/g. Based on the estimated cost/month and the evidence for efficacy and side-effects, topical steroids (fluocinonide > dexamethasone > clobetasol > triamcinolone) appear to be more cost-effective than topical calcineurin inhibitors (tacrolimus > pimecrolimus > cyclosporine) followed by intra-lesional triamcinolone.

Systemic steroids can require complex dosing schedules and carry an increased risk of side effects. They are most used short-term to treat severe flare-ups, and while low cost, monitoring and treating side effects when used longer term can significantly alter the cost-to-benefit ratio. Surprisingly, few trials have studied the use of systemic steroids in OLP, and only one comparing short-term betamethasone pulse therapy to topical triamcinolone met the inclusion criteria [67]. The average price of betamethasone 0.5 mg tablet is $0.58/tablet, but the total cost would vary according to the frequency and duration of the steroid pulse. Another systemic agent, dapsone which costs about $1.92/100 mg tablet was demonstrated to have increased efficacy over topical triamcinolone, tacrolimus, and retinoids [40].

There are several limitations to our study. There was significant heterogeneity in inclusion criteria and outcome measures of the RCTs included in this systematic review. Inclusion criteria of some trials required only a clinical diagnosis of OLP, while others required biopsy proven or symptomatic OLP. Furthermore, variable outcome measures, different trial durations, dosing regimens, and small sample sizes limited objective comparison of treatment outcomes. This heterogeneity underscores the necessity of developing consensus outcome measurements in the treatment of OLP to reduce study biases and allow for meta-analyses.

## Conclusion

Various therapeutics have been used for the treatment of OLP over the past five decades, but a consensus treatment guideline is still lacking. In this systematic review, topical steroids were found to be potentially the most economical and efficacious treatment modality followed by topical calcineurin inhibitors supporting the use of topical steroids as the first-line treatment with escalation to other treatment modalities only as needed. Future standardized RCTs and meta-analyses are required to assess the efficacy of additional therapeutics, especially systemic therapies.

## Supplementary Information

**Additional file 1: Table S1**. Consensus efficacy list of topical steroid and non-steroidal therapies.

**Additional file 2: Table S2**. Reported adverse reactions to oral lichen planus interventions.

## Data Availability

All data generated during this study are included in this published article (Table 1).
