# Supplementary material for: Oral lichen planus: comparative efficacy and treatment costs—a systematic review
Source: BMC Oral Health. 2022 May 6;22:161. doi: 10.1186/s12903-022-02168-4 (PMC9074269; doi:10.1186/s12903-022-02168-4)
Supplement: Supplementary file 1 — Additional file 1: Table S1. Consensus efficacy list of topical steroid and non-steroidal therapies. [file 12903_2022_2168_MOESM1_ESM.docx]

| ***Steroidal therapies***  *(Topical)* | ***No. of supporting RCTs*** |
| --- | --- |
| 1. Clobetasol   Gel 0.05%  Ointment 0.05% | 3  6 |
| 1. Betamethasone valerate   Cream 0.1% | 1 |
| 1. Dexamethasone   Solution 0.5 mg/5 ml | 7 |
| 1. Triamcinolone   Paste 0.1% | 12 |
| 1. Fluocinonide   Ointment 0.025% | 1 |
| 1. Fluocinolone acetonide   Ointment 0.025% | 1 |
| 1. Fluticasone propionate   Spray 0.05% | 1 |
| ***Second line therapies***  *(Topical/lesion directed)* |  |
| 1. Triamcinolone intra-lesional   10 mg/ml | 2 |
| 1. Tacrolimus   Ointment 0.1% | 9 |
| 1. Pimecrolimus   Cream 1% | 10 |
| 1. Cyclosporine   Solution 100 mg/ml | 5 |
| 1. Photo-dynamic therapy | 3 |
| 1. Low level laser therapy | 3 |
| 1. Aloe vera   Gel 70% | 2 |

Supplemental Table 1. Consensus efficacy list of topical steroid and non-steroidal therapies.
