# Supplementary material for: Oral lichen planus: comparative efficacy and treatment costs—a systematic review
Source: BMC Oral Health. 2022 May 6;22:161. doi: 10.1186/s12903-022-02168-4 (PMC9074269; doi:10.1186/s12903-022-02168-4)
Supplement: Supplementary file 2 — Additional file 2: Table S2. Reported adverse reactions to oral lichen planus interventions. [file 12903_2022_2168_MOESM2_ESM.docx]

| **Medication Class** | **Reported side effects** |
| --- | --- |
| ***Topical steroids*** |  |
| Clobetasol | Candidiasis, mildly elevated FBS, GERD |
| Triamcinolone | Candidiasis, GI upset, mucosal desquamation, tingling, burning, discomfort |
| Betamethasone | Candidiasis |
| Fluocinolone | Candidiasis |
| ***Topical calcineurin inhibitors*** |  |
| Tacrolimus | Discomfort, burning, tingling, sensitivity |
| Pimecrolimus | Burning, tingling, dysgeusia, xerostomia, GERD, herpes labialis |
| Cyclosporine | Burning, GI upset, lip swelling and itching, petechial hemorrhage, breast tenderness, dizziness |
| ***Other topical agents*** |  |
| Retinoid | Burning, dysgeusia, superficial mucosal desquamation |
| ***Intralesional therapies*** |  |
| Triamcinolone | Local erythema |
| BCG-PSN | Local burning, swelling |
| ***Systemic therapies*** |  |
| Retinoid (Etrentinate) | Skin and mucosal dryness, keratoconjunctivitis, rash, itchiness, hair loss, headache |
| ***Other procedure-directed therapies*** |  |
| Laser | None reported |
| PDT | Discomfort |
| Cryotherapy (nitrous oxide) | Local swelling |
| ***Natural alternatives*** |  |
| Lycopene | None reported |
| Ignatia | None reported |
| Aloe vera | None reported |

**Supplemental Table 2**. Reported adverse reactions to oral lichen planus interventions

FBS: fasting blood sugar, GERD: gastroesophageal reflux disease,
